# Recycled Cellulose Fiber Reinforced Plaster

**DOI:** 10.3390/ma14112986

**Published:** 2021-05-31

**Authors:** Nadezda Stevulova, Vojtech Vaclavik, Viola Hospodarova, Tomáš Dvorský

**Affiliations:** 1 Faculty of Civil Engineering, Institute of Environmental Engineering, Technical University of Kosice, Vysokoskolska 4, 04200 Kosice, Slovakia; viola.hospodarova@gmail.com; 2 Department of Environmental Engineering, Faculty of Mining and Geology, VSB-Technical University of Ostrava, 17. Listopadu 2172/15, 708 00 Ostrava-Poruba, Czech Republic; vojtech.vaclavik@vsb.cz (V.V.); tomas.dvorsky@vsb.cz (T.D.)

**Keywords:** waste paper fiber, fiber-cement plaster mortar, physical and mechanical performance, limestone, granulated blast furnace slag, adhesive strength

## Abstract

This paper aims to develop recycled fiber reinforced cement plaster mortar with a good workability of fresh mixture, and insulation, mechanical and adhesive properties of the final hardened product for indoor application. The effect of the incorporation of different portions of three types of cellulose fibers from waste paper recycling into cement mortar (cement/sand ratio of 1:3) on its properties of workability, as well as other physical and mechanical parameters, was studied. The waste paper fiber (WPF) samples were characterized by their different cellulose contents, degree of polymerization, and residues from paper-making. The cement to waste paper fiber mass ratios (C/WPF) ranged from 500:1 to 3:1, and significantly influenced the consistency, bulk density, thermal conductivity, water absorption behavior, and compressive and flexural strength of the fiber-cement mortars. The workability tests of the fiber-cement mortars containing less than 2% WPF achieved optimal properties corresponding to plastic mortars (140–200 mm). The development of dry bulk density and thermal conductivity values of 28-day hardened fiber-cement mortars was favorable with a declining C/WPF ratio, while increasing the fiber content in cement mortars led to a worsening of the water absorption behavior and a lower mechanical performance of the mortars. These key findings were related to a higher porosity and weaker adhesion of fibers and cement particles at the matrix-fiber interface. The adhesion ability of fiber-cement plastering mortar based on WPF samples with the highest cellulose content as a fine filler and two types of mixed hydraulic binder (cement with finely ground granulated blast furnace slag and natural limestone) on commonly used substrates, such as brick and aerated concrete blocks, was also investigated. The adhesive strength testing of these hardened fiber-cement plaster mortars on both substrates revealed lime-cement mortar to be more suitable for fine plaster. The different behavior of fiber-cement containing finely ground slag manifested in a greater depth of the plaster layer failure, crack formation, and in greater damage to the cohesion between the substrate and mortar for the observed time.

## 1. Introduction

Buildings have huge untapped potential to become a key part of the solution to urgent sustainability challenges. One of the important ways to improve the sustainability of buildings is to produce environmentally friendly materials and to design green composites for eco-friendly building constructions meeting the requirements of low-energy, zero-carbon green buildings with their structural components being highly thermally insulating and breathable, ensuring effective climatic control. In this context, the construction industry needs to take a circular approach considering the entire life cycle of its production chain [1].

Recently, significant efforts to develop new sustainable composite materials have been mainly related to the replacement of traditional cement binders in cement mortars and concrete products through alternative cementitious materials [2] and the utilization of natural renewable fibrous materials [3]. Cementitious-based composites containing cellulose fibers meeting the sustainability requirements have become increasingly important for economic, environmental, and technical reasons [3,4,5]. A driving force for using natural vegetable fibers as reinforcing materials in structural applications is their ecological nature, biodegradability, good mechanical properties, and ability to exhibit good tensile strengths in the composite [6,7,8,9,10]. The incorporation of plant-based fibers into cementitious composites has been found to be an effective and low-cost solution for improving the brittle nature of cementitious composites [11]. According to [12], plant fiber-reinforced cementitious composites have a lower environmental impact than traditional materials, require less industrial and technology transformations, and improve the life cycle assessment of the final product [13] when compared with classic or enhanced concretes [14]. However, the adhesion between the fiber and matrix plays a significant role in the final mechanical properties of the composites. The stress transfer between the matrix and fibrous filler determines the reinforcement efficiency [15]. As demonstrated in [16], poor fiber-matrix bonding, aggregation of fibers, and increased porosity lead to an inferior strength and stiffness for plant fiber-reinforced cementitious composites. Another dominating factor affecting the mechanical properties is their reciprocal compatibility, leading to a homogeneous distribution of the reinforcing fibers in the matrix [17].

The main progress in the current building sector lies in the use of cellulosic nano-/micro-fibers/-fibrils extracted from diverse plant non-wood sources, including hemp, jute, flax, sisal, bamboo, bagasse, date-palm, etc., into composite materials of high-quality performance, service ability, durability, and reliability standards [18,19,20,21,22]. This strategy also plays a significant role in increasing the mechanical performance of mortars. Cement mortars reinforced by plant fibers are widely studied for various uses in the construction of new buildings and in the restoration of historical structures. Their use as plaster—one of the important parts of construction, covering the outdoor and indoor surfaces of buildings—is conditioned by the development of such compositions with improved functional properties. While external plaster provides protection from weather effects, it mainly offers a protective surface against the penetration of rain water and other atmospheric agents, improves the appearance of the structure, and gives decorative effects; internal plasters, in addition to their aesthetic function, mainly ensure the best possible diffusion properties, and thus they allow for improving indoor air quality, positively impacting the health of the occupants [23].

In recent years, a dramatic increase in the incorporation of cellulose fibers into cement-based, lime-based, or gypsum-based plasters/mortars has been reported, in line with the trend of sustainability in construction [24,25,26,27,28]. Research efforts in this area have confirmed that the application of cellulose fibers in quasi-brittle matrices of plaster mortars remarkably improves their mechanical, thermal, and acoustic properties [29,30,31,32]. The minimization or prevention of cracks propagation as a result of their crack-bridging ability has been proven [33,34,35]. The addition of natural fibers in plaster strengthens the plaster coating and thus enhances ductility. Over the last decades, lignocellulosic or cellulosic fibers derived/extracted from various plants (abaca, coir, cork, flax, hemp, rice straw, and sisal) have been used in plaster mortars/composites [6,8,36,37,38,39,40,41,42,43]. The use of a combination of two natural fibers (kenaf and sisal) for the reinforcement of lime plaster was studied in [26]. The developed multifunctional plaster contained TiO_2_ nanoparticles (1 wt.%) and 0–4 wt.% cellulose fibers of two distinct sizes (1 mm < d < 2 mm; 2 mm < d < 4 mm), and showed superior thermal insulation properties, as well as the ability to control the indoor relative humidity and nitrogen oxide (NOx) photocatalytic degradation [44]. 

A key aspect of the application of lignocellulosic fibers in mortars is the nature of the inorganic matrix. As is known [45,46], the presence polymers like lignin and hemicelluloses in lignocellulosic fibers affected the setting process, and reduced the mechanical and durability properties of cement paste. Therefore, the pre-treatment of lignocellulosic fibers (hemp and flax) by chemical methods regarding the increase in the mechanical performance of lime and for mitigating the degradation of fibers in cement reinforced mortars was investigated [47,48,49]. As the quality of kraft fibers and pulp obtained through the chemical process of removing amorphous lignin and hemicelluloses from primary plant/wood sources is different from that of the cellulosic fibers obtained by recycling from secondary sources of waste paper, the integration of waste paper fibers (WPF) in the cement matrix requires more attention from researchers so that their usefulness can explicitly be examined. Waste paper containing a high amount of cellulose is an important source of cellulosic fibers, and because of the presence inorganic residues from the mineral fillers (calcite and kaolinite) used in paper making, is considered to be the most prominent recycled waste material, expanding the opportunities for its utilization in cement-based materials [50]; it supports the European Union circular economy goals [51,52]. 

As is shown in [24,53,54,55], the incorporation of waste paper fiber (WPF) has improved the properties of hardened cement-based composites. Thanks to the well-known homogeneity and composition of recycled fibers from waste paper, they are increasingly added in small amounts to cement mortars, providing a high-performance mortar/plaster with an insulating function and low environmental impact [56,57]. According to the results in the literature [58], the WPFs addition (0.2% by mass of cement) was found to show a significant self-shrinkage cracking control, while providing some internal curing. In [59], the effect of the addition of recycled cellulose fibers (up to 16 wt.% by mass of cement), obtained from waste packaging boxes and paper on the mechanical and thermal properties of lightweight cement paste was studied. The results revealed a reduction in the compressive strength of cement composite with the increasing fiber content; however, the thermal insulation properties were improved. Fibers derived from waste paper in various contents were applied in cement-based mortar mixed in a different way [25]. New ecological lightweight plastering mortars based on waste cellulosic fibers (newsprint or copy paper) using a technology with minimal embodied energy with very good thermal insulation characteristics, compressive strength assigned to class CS I or CS II, and water absorption by capillarity classified as W0 or W1 were developed [60]. Another effective approach to improving the properties of fiber-cement mortar is to partially replace the cement with supplementary cementitious materials (SCMs) [61,62]. SCMs positively influence the hydration kinetic of cement and lead to a considerable consumption of Ca(OH)_2_.

Our research was devoted to the extensive investigation of the properties of multiple types of kraft wood pulp and recycled fibers from waste paper [63,64], as well as their implementation into cement mortars [55,65,66,67,68,69,70]. As the valorization of waste paper fibers in cement mortars has been relatively unexplored in current literature, a research emphasis was placed on the identification of a suitable type of cellulose fibers from the six samples of wood pulp and waste paper fibers for their use in cement fiber reinforced mortar. The originality of this work is connected with the development of recycled fiber reinforced plaster mortar with a good workability for the fresh mixture, and insulation, mechanical and adhesive properties for the final hardened product for indoor application. The research focus was on studying the influence of the mass ratio of cement and waste paper fibers (C/WPF) in fiber-cement mortars containing three types of WPF (different in cellulose content and degree of polymerization) on technically important properties of fresh mixture and compact test specimens after 28 days of hardening. Another investigation was aimed at testing the adhesion of fiber-cement mortar with a modified composition to two commonly used substrates (brick and aerated concrete block). The specific goal of this work is to provide a comprehensive overview of the key findings related to the use of recycled cellulosic fibers in cement-based mortars and plasters.

## 2. Materials and Methods

### 2.1. Materials

The basic components of the fiber-cement mortar used here were as follows: Ordinary Portland cement CEM I 42.5 N, standard natural silica sand, tap water, and waste paper cellulosic fibers (recycled). The addition of a super plasticizer and thickener to selected mixtures was applied for an improvement in the workability and water retention. Other mineral substances such as limestone and blast furnace granulated slag were also applied into a set of fiber-cement mortars for testing their adhesion to the substrate.

#### 2.1.1. Cement

Portland cement CEM I 42.5 N supplied by Cement Factory Ltd. (Povazska Cementaren Ladce, Ladce, Slovakia) in accordance with the European standard [71] and was used in all of the cement mortar samples. Its chemical composition is in Table 1. 

The mean particle size of the cement (calculated from the particle size analysis data) was 24.03 µm, and the specific surface area value measured using the B.E.T method was 0.968 m^2^/g.

#### 2.1.2. Silica Sand

CEN standardized silica sand (fraction 0–2.0 mm) packed in bags (1350 g ± 5 g) supplied by Filtracni pisky (Chlum, Czech Republic) with a silica content of at least 98% as a filler in fiber-cement mixtures in accordance with the standard [71] was used. 

#### 2.1.3. Water

Tap water was used for the preparation and treatment of fiber-cement mixtures in accordance with the standard [72].

#### 2.1.4. Waste Paper Cellulose Fiber (WPF)

Three types of WPF (A, B, and C), different in chemical composition, provided by Greencel, Ltd. (Hencovce, Slovakia), were used in the cement mortars (Table 2).

Their gray color corresponded to the remaining inks used in newspaper printing. The samples contained two inorganic substances, namely calcite and kaolinite, which are used as mineral fillers in the paper-making process [64]. The crystallinity index, one of the most significant crystalline structure parameters of the studied WPF samples, ranged 41–51%, and the lowest value was reached with sample C. The polymeric characteristics of the main component of cellulose, indicating the average polymer chain length (DP—degree of polymerization) and distribution of its molecular weights in the cellulose polymer (PDI—polydispersity index), are summarized in Table 3. Both parameters are numerical outputs of the molecular mass distribution of the cellulose obtained using the size exclusion chromatography method. PDI represents the ratio of the mass-average molecular mass (Mw)/number-average molecular mass (Mn). DP was calculated by dividing the molecular mass by the monomer equivalent mass of anhydroglucose [64]. DP is an important structural parameter that significantly influences the mechanical properties of cellulose.

**Figure 1:**
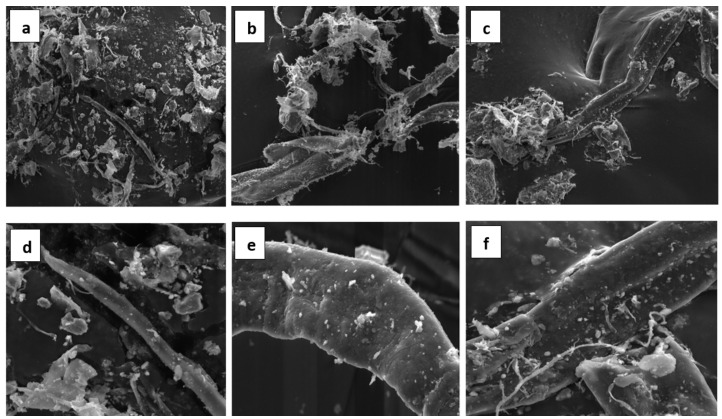
SEM micrographs of WPF samples (**a**,**d**) A, (**b**,**e**) B, and (**c**,**f**) C at magnifications of 1500× and 6000×.

The characterization of the selected physical properties of the WPF samples (a more detailed description of the methods for determining these properties is in paper [64]) is given in Table 4. The morphology of the fibers is shown in Figure 1. Heterogeneity on the fiber dimensions and the roughness of the fiber surface due to the presence of fiber fragments and impurities is clearly observed in the WPF samples. The fragments are formed as a result of the mechanical damage to the fibers due to the recycling process of waste paper. The impurities originated from the paper-making processes.

#### 2.1.5. Thickener

For the improvement and controlling of water retention, influencing the rheological properties, and adhesion of the fiber-cement mortar to the substrate, a thickener (mecellose) as a modified water-soluble cellulose ether derivative (hydroxypropylmethylcellulose (HPMC) manufactured by Lotte Fine Chemicals Ltd., Seoul, South Korea) in an amount of 1% by weight of the cement was used.

#### 2.1.6. Mixed Hydraulic Binders for Plastering Mortar

For experiments aimed at studying the adhesive ability of the fiber-cement mortar on the substrate, the binder component of the cement mortar was modified by using very fine ground products of natural limestone and metallurgical waste of granulated blast furnace slag (from KOTOUČ ŠTRAMBERK Ltd., Štramberk, Czech Republic). The fine products of the granulated slag as a powdered latent hydraulic binder met the requirements of the standard for [73], while ground limestone complied with the requirements of the standard for [74]. The chemical composition of both mortar components is in Table 5. The mean particle diameter of both mortar components was almost the same, 12.82 µm and 12.25 µm, respectively; however, the specific surface area of the ground slag was lower (0.96 m^2^/g) than the value corresponding to the ground limestone (1.46 m^2^/g).

### 2.2. Preparation of Fresh Fiber-Cement Mixtures

Two sets of experimental cement mortar mixtures with different contents of all WPF samples, expressed as the cement to WPF material mass ratios (C/WPF), were prepared (I and II), as shown in Table 6. Based on the results of testing the fresh mixtures and hardened mortars of the sets, fiber sample C as a sand replacement and representative of the group of WPF with the highest content of cellulose and degree of polymerization was selected for the production of fiber-cement plaster mortars (set III) with a modified binder composition (see Section 2.1.6). The addition of 1 wt.% thickener (mecellose) was used in the mortars. The water to cement ratio (W/C) was varied because of the changing amount of fibers used in the mixture and taking into consideration the hydrophilic nature of WPF (Table 6). 

The design of the composition of the sets of fiber-cement mortars was based on the standard for [71]; the mass ratio of cement/sand (C/S) was 1:3. Control (reference) cement mortars (R1, R2, and R3) without WPF addition were prepared. R1 was prepared according to the standard with W/C = 0.5. Reference samples R2 and R3 with varied W/C ratios corresponding to fiber-cement mortars were prepared so as to compare the properties of the fiber-cement mortars. The mass ratio of the components in the mortar mixture of set III (cement:limestone (slag):WPF:mecellose) was equal 1:2.04:0.17:0.01 (mortar based on limestone (C8) and slag (C9), respectively). The preparation of fiber-cement mixtures (I-II) consisted of two steps. The soaking of recycled fibers and manual mixing in approximately 50 wt.% of water was the first step in the preparation of the mixture. Next, the remaining water, the required amount of sand, and the cement were mixed by mechanical stirring in a mixer. Mixing took place automatically in a laboratory mixer BS MI-CM5AX (BETON SYSTEM, Brno, Czech Republic), according to the standard for [71] for 5.5 min. The procedure for preparing fiber-cement plasters (III) was as follows: the weighed individual dry materials (cement, limestone/slag, and WPF) were mixed, and water was gradually added during mechanical stirring. After sufficient mixing of the components, a thickener was added and incorporated into the mixture.

### 2.3. Production of Test Specimens

Standard steel molds with dimensions of 40 × 40 × 160 mm were used for the production of the mortar test specimens. The filling of the molds with fresh fiber-cement mixtures (I and II) took place in two layers; each layer being compacted by 60 strokes of the compaction table (according to the standard for [71]). The compaction of each layer of mixtures (set III) was performed by tapping (20 times) from a height of 3 cm on both sides of the mold. The excess layer of the mixture was wiped off with a saw motion and smoothed with a metal ruler. The filled molds were covered with PVC foil, which served to prevent water evaporation from the fresh mixture needed to hydrate the cement. Curing of these mix sets (I and II) took place for 2 days in molds at room temperature and humidity (+20 °C; 50% RH). After that time, the bodies were removed from the molds and were placed in a water bath (sets I and II), and the fiber-cement mixes (III) were held under PVC foil in a laboratory for 26 days.

In total, about 800 test specimens were prepared for testing the properties of the fresh fiber-cement mixtures and the technically significant parameters of the composites. Errors bars in the histograms represent the standard deviation of the three replicates.

### 2.4. Methods for Testing Properties of Fresh Mixtures and Compact Specimens

#### 2.4.1. Consistency

The flow behavior of the fresh fiber-cement mixtures (indicating its workability) was detected through the determination of consistency using a flow table test in accordance with the standard of [75]. The mean diameter of a test sample placed on a flow table was measured before being impacted vertically after the release of a standard slump cone. The measurement was repeated twice with an accuracy of ±1 mm. 

#### 2.4.2. Physical Properties of Compact Specimens

All of the physical properties of the compact mortar specimens were measured on prismatic bodies after 28 days of hardening.

##### Dry Bulk Density

Dry bulk densities of the 28-day hardened fiber-cement composite specimens were calculated according to the standard for [76].

##### Water Absorbency and Capillary Water Absorption

The water absorbency of mortars was determined according to [77]. The weighed specimens were immersed in a water bath at room temperature (20 °C) until their weight stabilized. The composites were then weighed and placed in an oven where they were dried at 80 °C until reaching a constant weight. 

**Figure 2:**
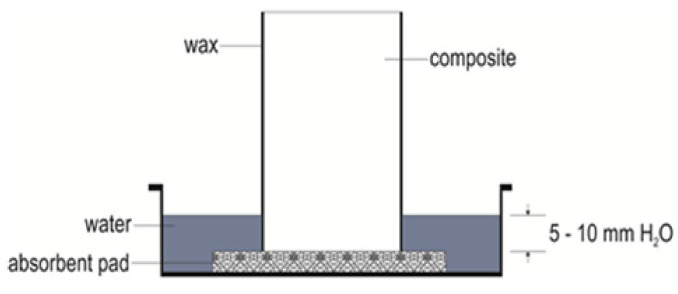
Capillary water absorption testing scheme.

The standard for [78] was used for the determination of the capillary water absorption coefficient of the hardened fiber-cement mortars. A sealing compound (paraffin wax) was applied to the longitudinal sides of the test prisms, and they were broken in two halves. The prismatic bodies that were prepared were inserted with the fracture surface downwards into a container with water, the level of which was 5–10 mm (see Figure 2). Washers were inserted between the bottom of the vessel and the test specimen (its fracture surface) to ensure perfect contact with the water. The water level was kept constant throughout the test. The capillary water absorption coefficient (C) was determined from the difference of the two weight values measured after 10 (M1) and 90 min (M2), according to Equation (1)

(1) $$C=0.1\times \left(M2-M1\right)$$

The resulting average value of C is given with an accuracy of 0.05 kg/m^2^·min^0.5^.

##### Thermal Conductivity

The coefficient of the thermal conductivity of the hardened reference samples and fiber-cement mortar specimens was measured on their surface using the commercial device ISOMET 2114. The tests were performed using a measuring surface probe IPS 1100 placed on the surface of each wall of the prismatic specimen, and the mean value was reported. 

#### 2.4.3. Mechanical Properties of Compact Specimens

The compressive strength of the fiber-cement mortars (40 mm × 40 mm × 160 mm) after 28 days of hardening was determined using a compression test machine (FORM + TEST Seidner and Co. GmbH, Riedlingen, Germany) with a maximum load force of 300 kN and a loading rate of 2400 ± 200 N/s, in accordance with [79]. A three points bending test was used for the determination of the flexural strength on the aforementioned testing machine, with a maximum load force of 10 kN and a loading rate of 50 ± 10 N/s. The resulting parameter values were the average of six measurements.

#### 2.4.4. Adhesive Strength of Plastering Mortars on the Substrates

**Figure 3:**
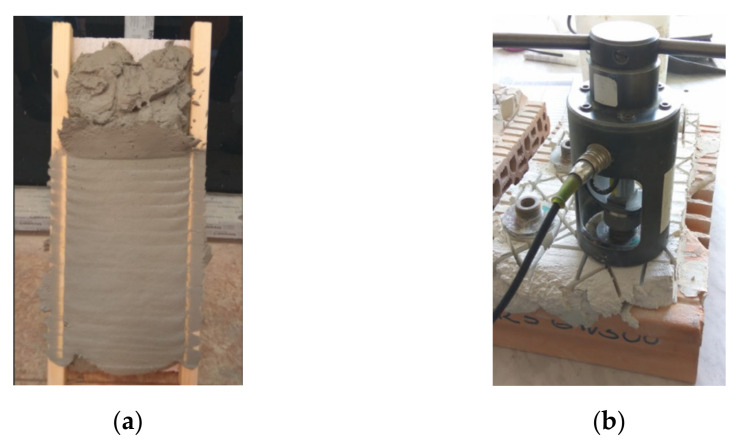
Application of fiber-cement mortar on the substrate of an aerated concrete block (**a**); demonstration of testing the adhesion of fiber-cement mortar to the substrate (**b**).

The adhesive strength of the hardened plaster cement mortars based on the WPFs for two selected substrates (brick and aerated concrete blocks) was measured according to the standard for [80], which expresses the maximum tensile stress derived from a load perpendicular to the surface of the mortar applied to the substrate. The tensile load was determined using a Comtest OP3/4 (COMING Plus Ltd., Prague, Czech Republic) and a rigid tear-off target (diameter of 50 ± 0.1 mm; height at least 10 mm) made of corrosion-resistant steel, and it adhered to the tested surface of the mortar surface with Sikadur-31 CF Rapid adhesive (Sika Slovakia, Ltd., Bratislava, Slovakia), according to standard [80]. The mortar sample in a thickness of 30 ± 1 mm was applied to the substrate of ceramic fittings (375 mm × 80 mm × 238 mm) and aerated concrete blocks (50 mm × 249 mm × 599 mm). The substrate was kept vertical during application. The specimens were tested after 28 days of hardening. Five test circular surfaces with a diameter of 50 mm were drilled into the mortar so that the substrate was cut to a depth of at least 2 mm. Tear-offs were performed using glued test targets. The testing of the adhesion of the mortar to the substrate is demonstrated in Figure 3. The adhesion R_fu_ (in MPa) is expressed by Equation (2) as the ratio of the derived load F (in N) and test area A (mm^2^).

(2) $$R_{\mathrm{fu}}=F/A$$

## 3. Results and Discussion

### 3.1. Effect of C/WPF Ratio on Properties of Fresh Mixtures and Hardened Fiber-Cement Mortars 

#### 3.1.1. Consistency of Fresh Fiber-Cement Mixtures

**Figure 4:**
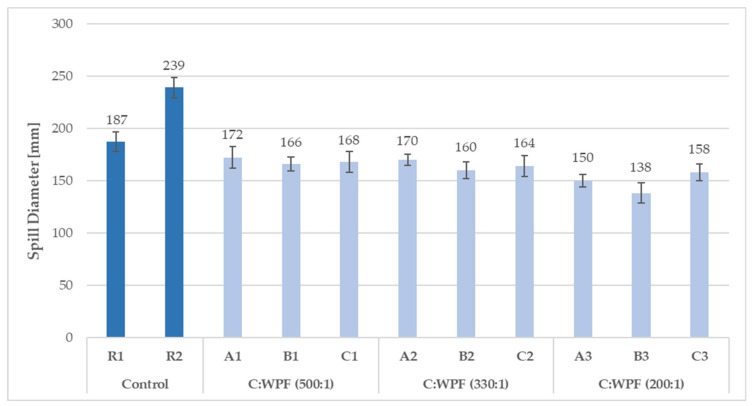
Mean spill diameter values for reference and fiber-cement fresh mixtures based on WPF samples (A, B, and C) with different C/WPF ratios (set I).

**Figure 5:**
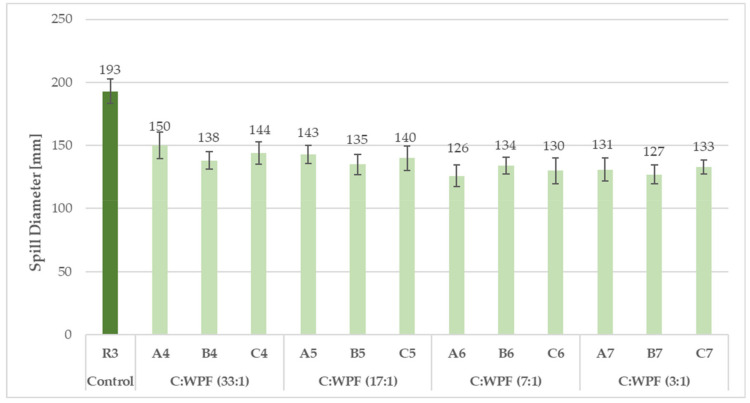
Mean spill diameter values for reference and fiber-cement fresh mixtures based on WPF samples (A, B, and C) with different C/WPF ratios (set II).

**Figure 6:**
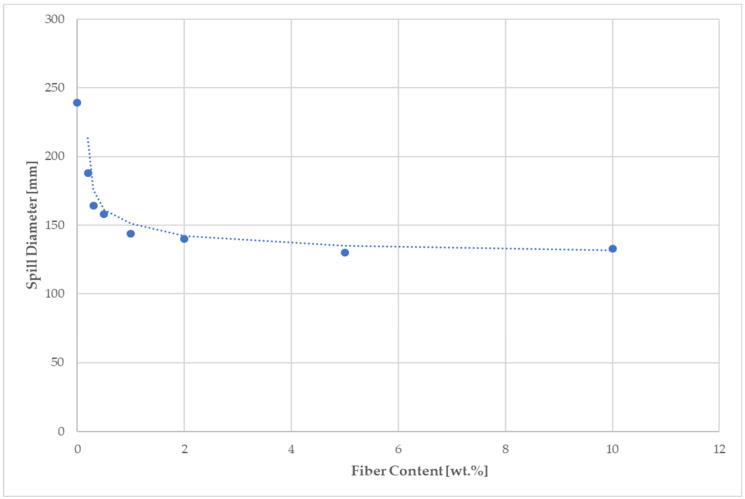
Effect of fiber content (sample C) on the consistency of the fiber-cement fresh mixture.

The consistency of fresh cement mixtures plays a vital role in the determination of the workability for mortars and for the compressive strength of hardened mortars. The water to cement ratio as a parameter determining the normal consistency of mortars and ensuring a complete hydration reaction was necessary in order to modify, in the case of fiber implementation into a cement mixture, in order to make a paste with an appropriate plasticity (Table 6). The effect of the WPF content in the cement mixes (expressed by C/WPF ratio) on the flow behavior of fresh fiber-cement mixtures was investigated. As shown in Figure 4, Figure 5 and Figure 6, a decrease in the resulting mean values of the spill diameter of the fresh fiber-cement mixtures with an increasing amount of cellulose fibers in the mixture and decreasing C/WPF ratio can be observed. This phenomenon corresponds to properties of incorporated WPF as a material with a rougher surface, voids, high water absorption ability, and low apparent density, which significantly affect the mortar consistency. Fibers absorb larger amounts of mixing water and consequently significantly reduce the resulting consistency of the fresh mixture [48]. All of the experimental fiber-cement mortars reached mean spill diameter values in the range of 188–133 mm. The course of the mean spill diameter values with an increasing fiber content (0.2–10 wt.% from weight cement) for sample C (the highest cellulose content) clearly demonstrated their strong reduction in consistency (Figure 6). We used a simple moving average for the spill diameter as a tool of technical analysis to identify the trend. According to the measured values of consistency, fiber-cement mortars with a fiber content ≤2 wt.% can be classified as plastic mortars (140–200 mm). This value represents the limit for the fiber content in fiber-cement mortars in terms of their good workability. A 5–7% loss of workability from the lower limit (140 mm) was recorded for mortars with an increasing content of short fibers (C/WPF of 7:1 and 3:1). The presented results demonstrate the role of fibers in inducing the plasticity and reducing the flowability of fresh mortars. Similar results have been reported for paper [17,81].

#### 3.1.2. Bulk Density and Thermal Conductivity 

**Figure 7:**
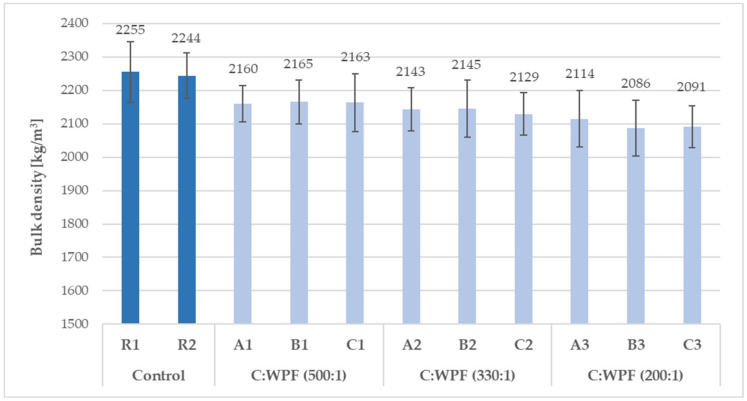
Development of bulk density with C/WPF ratios in fiber-cement mortars (set I) after 28 days of hardening.

**Figure 8:**
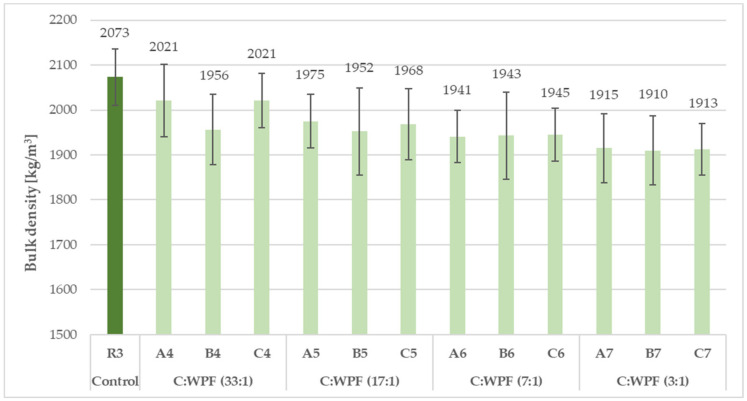
Development of bulk density with C/WPF ratios in fiber-cement mortars (set II) after 28 days of hardening.

**Figure 9:**
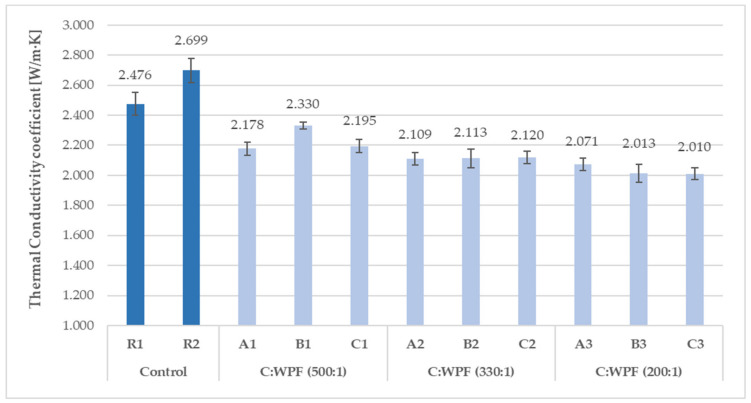
Thermal conductivity coefficients for 28-day hardened fiber-cement mortars (set I) with different C/WPF ratios.

**Figure 10:**
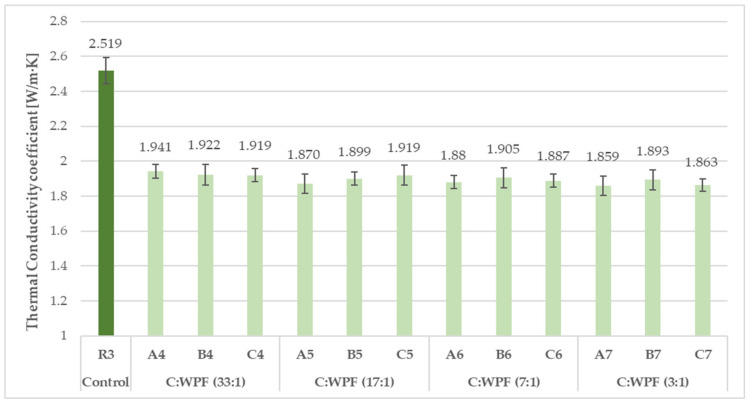
Thermal conductivity coefficients for 28-day hardened fiber-cement mortars (set II) with different C/WPF ratios.

From a comparison of the results of the bulk densities and thermal conductivity coefficients of fiber-cement mortars after 28 days of hardening composites (Figure 7, Figure 8, Figure 9 and Figure 10), it is clear that as the content of cellulose fibers in the mixture increased (C/WPF ratio decreased), the bulk density and conductivity of the mortars had a decreasing trend. The values of the bulk density ranged 2160 to 1955 kg/m^3^, and thermal conductivities were found in the range of 1.863–2.330 W/m·K. The values of both parameters found for references samples R1, R2, and R3 under the same laboratory conditions were likely mainly influenced by the ongoing phase transformation of the cement paste from a more liquid state to a solid state of mortar, and by the microstructure of hydrated phases formed under the conditions with different W/C ratios in the mixture during the hardening process. Another factor influencing the thermal conductivity can be related to the quality of the thermal contact of the measuring element and measured object. 

**Figure 11:**
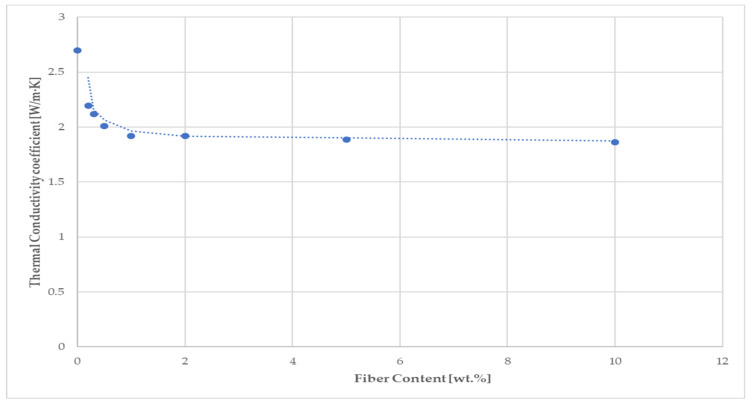
Dependence of the thermal conductivity coefficient of 28-day hardened fiber-cement mortars on the increased content of fiber sample C.

A comparison of the thermal conductivity values of the fiber-containing mortars with those found for the reference mortars showed an almost equal reduction by 24–35% due to the low apparent density of the cellulose fibers and the increasing proportion of fibers in the mixture. Based on the values of the bulk densities of the mortar samples in the dried state, they belonged to the ordinary mortars [82] without thermal insulation properties. The measured thermal conductivities for 28-day hardened reference mortars R2 and R3 (2.519 and 2.699 W/m·K, respectively) were very close to the value (2.7 W/m·K) found for the cement mortar in [83]. Figure 11 illustrates the trend in the simple moving average of the thermal conductivity coefficient for hardened mortar samples with an increasing content of fiber sample C in the mixture. Using recycled cellulose fibers led to a better thermal insulation behavior for the cement matrix. The best thermal conductivity (1.783 W/m·K) among the tested mortars based on fiber sample C was reached with 10 wt.% (C7). The short cellulose fibers bring more pores and cavities into the cement matrix, which leads to a reduction in the thermal conductivity of the samples. A similar behavior has been observed on cement composites based on recycled waste paper and paper packaging [59], and waste fiber from young coconut and durian [84]. 

As is known, the thermal conductivity of a material is determined not only by its chemical composition and structure, but also by the porosity, the nature of the pores, the moisture content of the material, and the temperature at which heat is transferred. The porosity of the fiber-cement material plays a crucial role, not only in thermal conductivity, but also in its water behavior.

**Figure 12:**
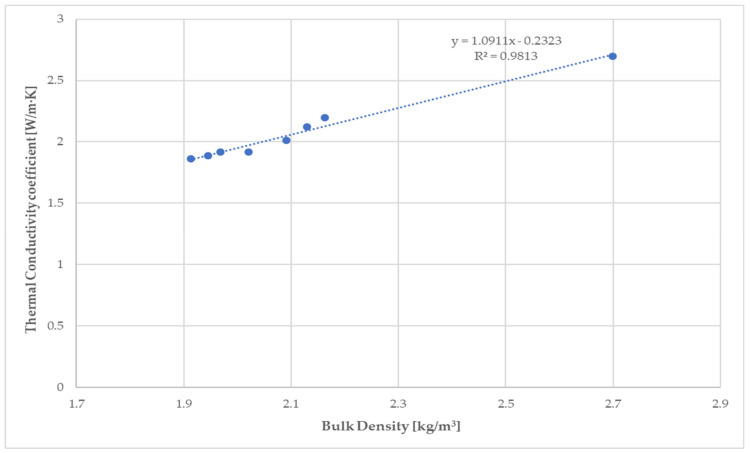
Relationship between the thermal conductivity coefficient and bulk density of 28-day fiber-cement mortar sample C with different C/WPF ratios.

The high value of the correlation coefficient (R = 0.9906) for the dependence shown in Figure 12 confirms the linear relationship between the thermal conductivity coefficient and the bulk density of the studied fiber-cement mortar of sample C with different C/WPF after 28 days of hardening. 

#### 3.1.3. Water Absorbency and Capillary Absorption 

As shown in Table 7, an increase in the absorbency of 28-day hardened fiber-cement mortars by 6.3–21.1% and 17–32.4% dependent on the WPF content was observed in comparison with the absorbency value of the control samples R2 and R3. The values of the capillary water absorption coefficient of the cement mortars containing recycled fibers ranged from 0.21 to 0.39 kg/m^2^·min ^0.5^ dependent on the C/WPF ratio, and were also higher than the value of the control samples. The water behavior of the fiber-cement mortars is of a complex nature and is affected by a number of factors [85]. The main cause of such a water behavior for fiber-cement mortars is the implementation of cellulosic fibers to the matrix of cement mortars, with their typical absorbency ranging from 7% to 20% [86].

The fiber content in the matrix significantly affects the formation of the pore structure in these materials. Therefore, water absorption is considered a measure of the porosity of fiber-cement composites/mortars, and provides useful information on the permeable volume of pores inside the sample and the potential interconnection between these pores [33]. It is worth noting that the volume of voids and the number of open and closed pores and their size significantly affect the water absorption of fiber-cement materials. 

**Figure 13:**
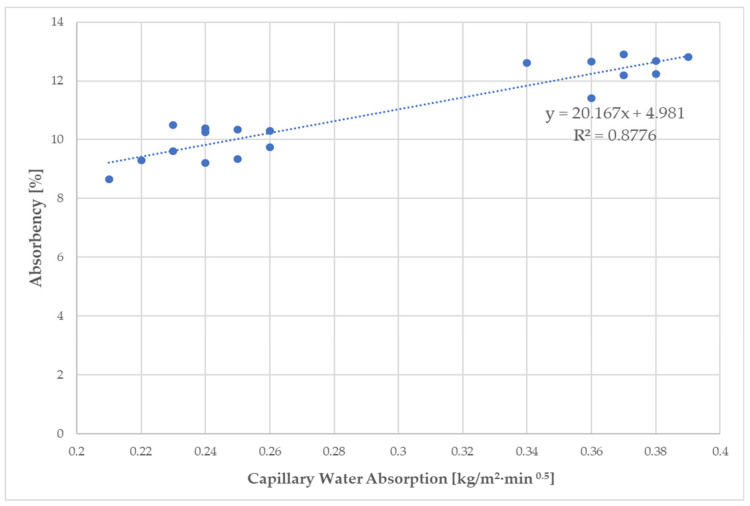
Relationship of the absorbency and capillary water absorption of fiber-cement mortars with increasing fiber content.

**Figure 14:**
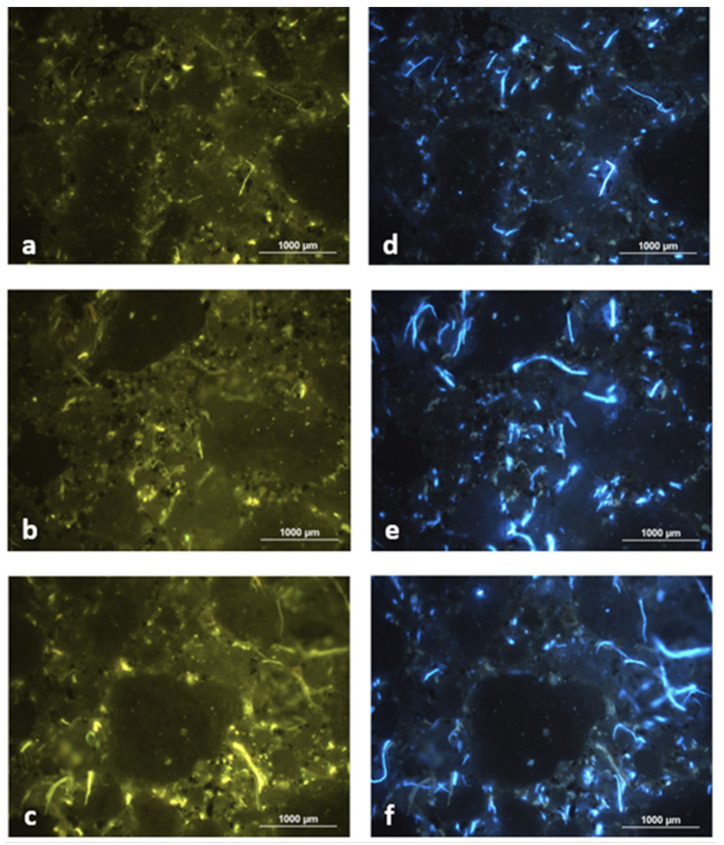
Micrographs of the texture of 28-day fiber-cement mortars samples A3 (**a**,**d**), B3 (**b**,**e**), and C3 (**c**,**f**) for C/WPF with a ratio of 200:1 under UV light using WB (left) and WU filter (right).

Figure 13 shows the linear relationships between both parameters of water behavior for fiber-cement composites based on a particular type of cellulose fibers with changing the C/WPF ratio. All of the measured values of capillary water absorption correspond to class W1 (≤0.40 kg/m^2^·min^0.5^), in accordance with standard for [82]. The lower fiber content in the cement mortar led to lower values of absorbency in the range of capillary absorption of 0.21–0.25 kg/m^2^·min^0.5^ because of the low porosity of the fiber-cement mortar, as well as the fibers themselves, while a higher fiber amount in cement paste (higher porosity) at almost equal capillary absorption values caused a higher absorbency. This relation between the measured values is most likely related to the number of open pores with different sizes, whereas the number of closed pores probably appears to be constant because they hardly exert capillary forces. Another possibility of this behavior could be related to the very small pore sizes in the mortar structure into which water molecules do not penetrate. As is evident from Figure 13, incorporating a higher proportion of WPF into mortars led to significantly higher values for both quantities, where the capillarity absorption values shifted to values higher than 0.26 up to 0.40 kg/m^2^·min^0.5^, and are related to the formation of a larger number of pores and to their size. Figure 14 documents the texture of the pores formed in fiber-cement mortar samples A3, B3, and C3 (C/WPF = 200:1). The pores are evenly distributed in the matrix and only occasionally are small agglomerates and clumps of fibers observed. The size of the pores, which are mostly spherical in shape, ranges from a few tenths of a millimeter to a millimeter. A small number of pores have an irregular shape, being slightly elongated. In some cases, the pores may be filled with fibers or clumps of fibers.

#### 3.1.4. Compressive and Flexural Strength 

**Figure 15:**
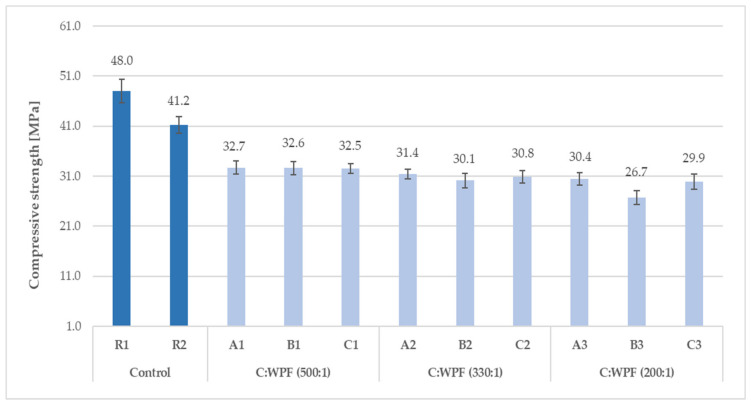
Compressive strength of fiber-cement mortars with different C/WPF ratios (set I).

**Figure 16:**
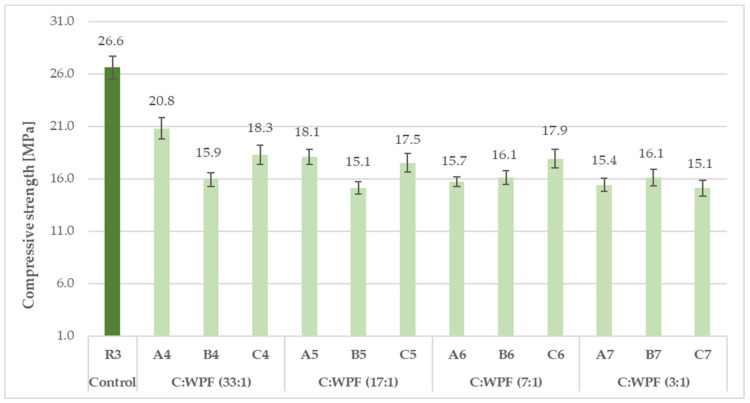
Compressive strength of fiber-cement mortars with different C/WPF ratios (set II).

**Figure 17:**
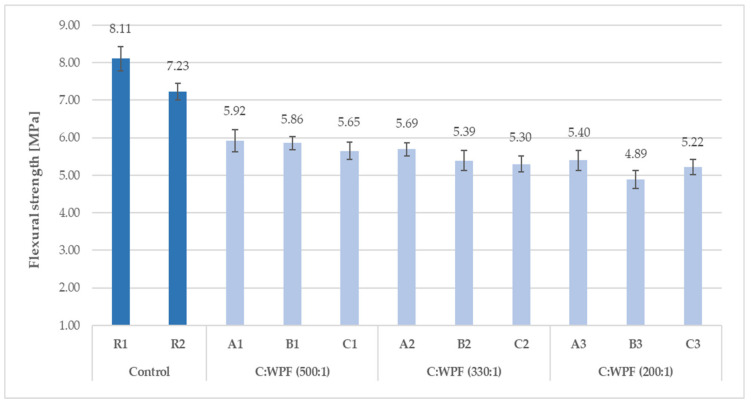
Flexural strength of fiber-cement mortars with different C/WPF ratios (set I).

**Figure 18:**
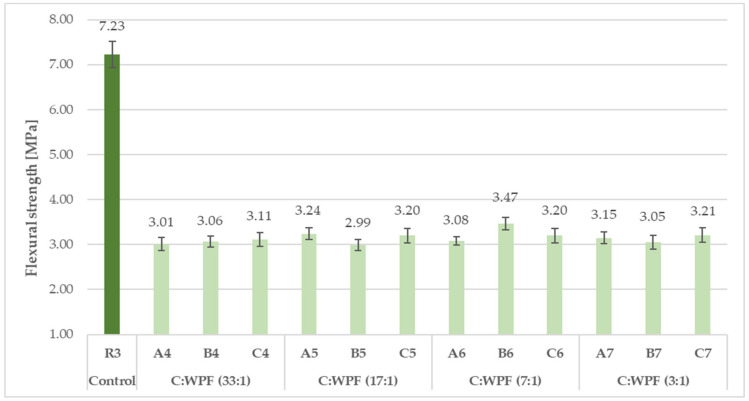
Flexural strength of fiber-cement mortars with different C/WPF ratios (set II).

The results of the compressive and flexural strength of the fiber-cement mortars with varied amounts of fiber content after 28 days of hardening are illustrated in Figure 15, Figure 16, Figure 17 and Figure 18. The C/WPF ratio had a significant effect on both of the strength characteristics of the mortars. 

**Figure 19:**
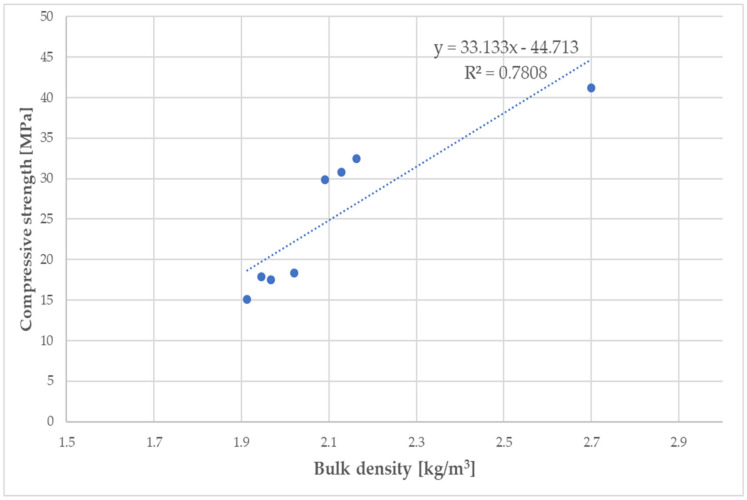
Linear dependence of compressive strength on bulk density of fiber-cement mortar samples (C1–C7).

As the C/WPF ratio decreased, the values of the compressive and flexural strength of the fiber-cement mortars decreased significantly. The fiber-cement mortars reached values of compressive and flexural strength in the range of 15.1–32.7 MPa and 3.01–5.92 MPa, respectively, depending on the WPF content, and they represented a lower level of the strength parameter found for reference samples R2 and R3. The higher the bulk density, the higher the value of the compressive strength of the fiber-cement mortar depending on the WPF content of sample C is observed (Figure 19; R = 0.8836).

**Figure 20:**
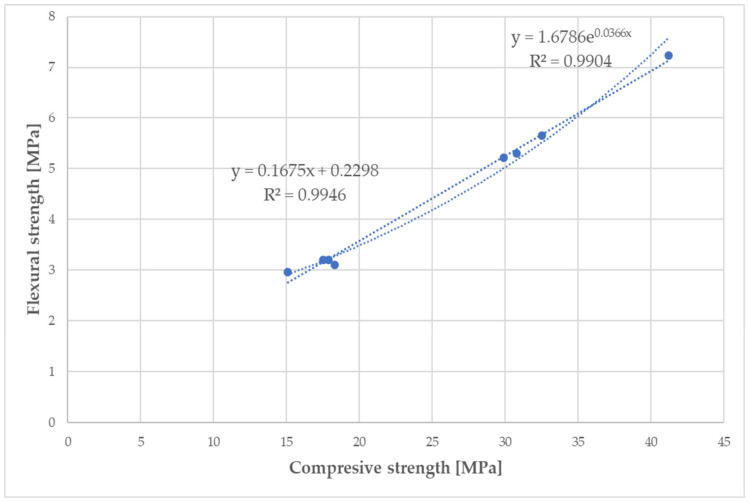
Relationship between flexural and compressive strength of fiber-cement mortars (C1–C7).

A slightly higher correlation coefficient was calculated for the linear dependence between the flexural strength and bulk density (R = 0.8927). The presented decreasing trends of both strength parameters with an increasing fiber content for the mortar samples (C1–C7) were observed. A higher fiber content in the cement mortar induces the formation of larger interfacial pores/voids that make a more lightweight material, and it should be pointed out that a weaker adhesion at the matrix-fiber interface owing to the hydrophilic nature of WPFs leads to a decrease in the compressive and flexural strength. Similar conclusions were also found by Bentchikou et al. [59] upon investigation of the effect of recycled waste paper fiber contents on the mechanical properties of lightweight cement composites. As shown in Figure 20, there is a proportionality between the experimental determined levels of flexural and compressive strength for fiber-cement mortar samples (C1–C7). Two equations (linear and exponential) with almost equal values for the correlation coefficients (0.9973 and 0.9952) were found for the relationships of these strength parameters. This means that both models included factors affecting the strength of the fiber-cement mortars after 28 days in the range of the measured strength levels, and the flexural and compressive strengths had a very good correlation. 

### 3.2. Modification of Fiber-Cement Plastering Mortar Composition and Its Influence on Technically Important Properties 

In terms of determining the practical implementation of recycled cellulose fibers into cement plastering mortar for internal use and improving its plasticity behavior, two very finely ground materials, namely, limestone and granulated blast furnace slag, were selected. The effect of the modified composition of the fiber-cement plastering mortar on the important parameters of the fresh mixtures and hardened mortars was investigated. 

#### 3.2.1. Physical Properties of Fresh Fiber-Cement Mixtures and Hardened Mortars

Table 8 summarizes the results obtained from the measurement of the consistency of fresh fiber-cement mixtures with the addition of very finely ground binder components of limestone (C8) and granulated slag (C9), and other properties of hardened mortars for application as interior plasters. As can be seen, the consistency values of the fiber-cement mixtures and the properties of the hardened specimens were influenced by the nature of the fine components in the cement mixture. The mean spill diameter of the fresh fiber-cement mixture with the addition of limestone (C8) reached a slightly higher consistency value than such a mixture with a slag addition (C9). This showed the same plasticity for both mortars.

The bulk density value of the hardened mortar with finely ground limestone is evidently related to its lower density (2710 kg/m^3^) compared with cement mortar containing granulated blast furnace slag (3100 kg/m^3^). The thermal conductivity coefficient values of both mortar samples were almost the same (0.224 W/m·K and 0.222 W/m·K), and they were almost at the same level as the data (0.28 W/m·K) for fiber-cement mortar containing 16 wt.% fiber published in [59]. The measured conductivities were significantly lower than those declared for lime (0.9 W/m·K), lime-cement (1.0 W/m·K), and cement plaster (1.2 W/m·K). 

Although the porosity of fiber-cement mortar plays a decisive role in this case, the different nature of the mineral components and the presence of waste paper fibers in the cement mixes affected the water absorption behavior of the mortar specimens (C8 and C9). The measured values of the absorbency and capillary water absorption were most likely related to the number of closed pores, which hardly exerted capillary forces; therefore, sample C9 reached lower values. As above mentioned, the water behavior of fiber-cement mortars is of a complex nature and is determined by a number of factors. Therefore, our assumption of a lower porosity for the C9 sample was not reflected in the thermal conductivity values.

#### 3.2.2. Mechanical Properties of Fiber-Cement Mortars 

The results of the compressive and flexural strength of fiber-cement mortars containing two components after 28 days are presented in Table 8, and showed two times higher values (10.07 MPa and 3.32 MPa, respectively) for both strength parameters for mortar C9 than for sample C8 (4.86 MPa; 1.70 MPa). The values of compressive strength found for the fiber-cement mortar samples of C8 and C9 corresponded to the minimum strength value required for the mortar of the CSIII (5 MPa) and CSII (2.5 MPa) classes [82]. The measured values for the flexural strength of the mortar samples were higher than the approximate flexural strength after 28 days of CS III (0.7 MPa). As the strength of such fiber-based materials depends on several factors, a higher value for the strength parameter (C9) could be related to a lower proportion of pores in the material. Another reason among the other factors influencing the strength of fiber-cement mortar might be attributed to better filling of the interparticle spaces in the mortar structure because of the presence fine particles of slag. Certainly, the chemical composition of this latent hydraulic material (CaO/SiO_2_ = 1.43) significantly contributes to the strength, unlike fiber-cement mortar with fine limestone. An unreasonably high CaO/SiO_2_ ratio in limestone (67.3), not supporting C-S-H phase formation in cement mortar, resulted in a decrease in strength properties.

#### 3.2.3. Adhesive Strength of Fiber-Cement Plastering Mortar on Substrates 

The adhesive strength of plaster mortar on a substrate (brick, concrete, and masonry) like the base layer is one of the important criteria for assessing the material quality, as well as its execution [87]. The plasticity of mortar directly depends on its ability to hold water against the suction of the surface to which it is applied. 

**Figure 21:**
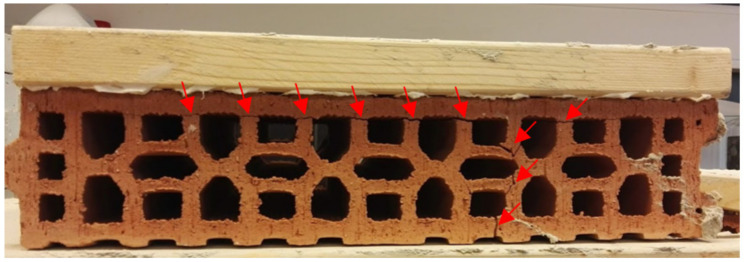
Damage of the brick substrate with C9 plaster mixture.

**Figure 22:**
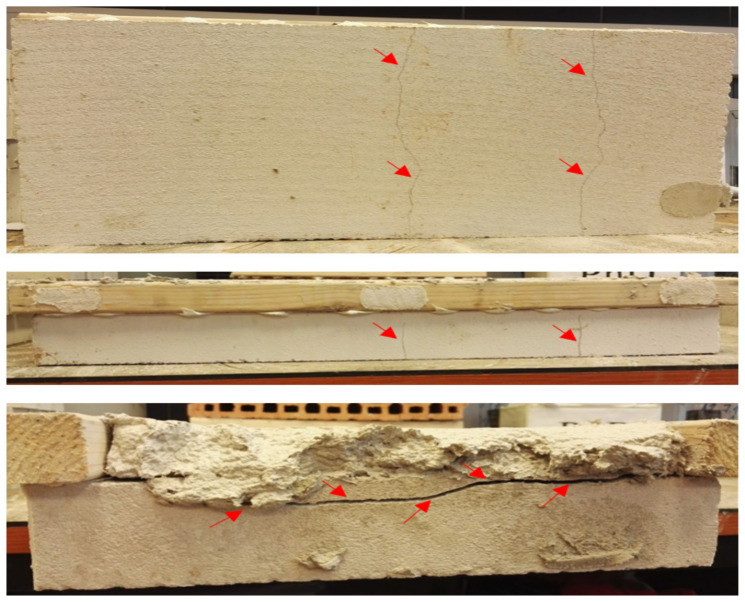
Damage of an aerated concrete block substrate with the C9 plaster mixture.

Testing the adhesion of the fiber-cement plastering mortars with finely ground limestone (C8) and granulated blast furnace slag (C9) on two absorbent substrates of brick (B) and aerated concrete blocks (ACBs) after 28 days indicated higher values of this parameter for the fiber-cement formulation containing finely ground slag (C9) on both substrates (0.347 MPa and 0.228 MPa, respectively) compared with the cement mortar sample based on finely ground limestone (0.215 MPa and 0.205 MPa, respectively). Our results exceeded the minimum adhesive strength of the mortar to the substrate (0.18 MPa) declared by the manufacturer in the technical data sheets. According to the standard for [79], the failure of mortar adhesion on the substrates was also monitored. The failure occurred mostly in the surface of the plaster layer applied to the substrate. Different failure depths for the C8 and C9 plaster mortar samples on the two substrates after 28 days were found. While this depth was the same, about 0.5 mm, for the C8 mortar on both substrate surfaces (B and ACB), mortar sample C9 had a breakdown depth ranging 22–43 mm [69]. In addition, the cohesion between the substrate and the plaster mortar was broken, cracks formed, and the substrates were finally damaged, as illustrated in Figure 21 and Figure 22. The damage of substrates (crack formation) was probably caused by the volume changes of the very finely ground granulated slag used in the proposed formulation (C9) because of its high tensile stress. 

These deficiencies caused the formation of cracks in the plaster mortars and the subsequent damage to the brick and aerated concrete block substrates due to applied layer of plaster mortar formulations, which could be eliminated by modifying the recipes to better fix the amount of water needed for hydration, as well as for processing fresh mixes in the mix. 

## 4. Conclusions

This work is focused on the valorization of waste paper fibers in cement mortars presenting an economic, technically feasible, and ecological approach to waste management and promoting a cleaner environment. The study presented the performance of sustainable cement-bonded mortars containing cellulose fibers from waste paper recycling. Based on the research results presented in two parts of this work, which were aimed at studying the effect of waste paper fiber content expressed by the cement to waste paper fiber (C/WPF) mass ratio on the important properties of fresh fiber-cement mixtures and 28-day hardened mortars, as well as a comparative investigation of the adhesive strength of fiber-cement plasters based on finely ground limestone and slag with two commonly used substrates, the following conclusions can be drawn:

1. The C/WPF ratio had a significant effect on all of the studied properties of fresh mixtures and hardened fiber-cement mortars. The consistency values of the fiber-cement mortars containing fibers with different cellulose contents in amounts less 2 wt.% were related to the plaster mortars. The optimum C/WPF ratio for achieving values of a mean spill diameter corresponding to the standard plastic mortars (140–200 mm) within the scope of the study is ranged up to 17:1, representing a fiber content ≤2 wt.% in cement mortar with fiber sample C. The higher fiber content in cement mortar and the higher porosity meant that the mortar became lighter, leading to lower values for the bulk density and thermal conductivity after 28 days of hardening. The study of the water absorption behavior of the fiber-cement mortars confirmed that incorporating a higher portion of WPF into mortars led to significantly higher values of absorbency and a capillarity absorption coefficient. This fact is related to the formation of a larger number of pores and their sizes because the low C/WPF ratio in cement mortars caused a decrease in the compressive and flexural strength. This is also a consequence of the weaker adhesion of the fibers and cement particles at the matrix–fiber interface because of the hydrophility of the cellulose fibers.
2. The influence of the fiber-cement mixture composition according to its modification of finely ground limestone and granulated slag in terms of achieving a better plasticity and satisfactory adhesive strength for two absorbent substrates (brick and aerated concrete block) was investigated. Monitoring the consistency and the physical and mechanical properties of 28-day hardened fiber-cement plastering mortars, as well as the adhesion of these mortars based on the high content of fiber type C (C/WPF = 6:1) for both substrates showed their different behavior, which was influenced not only by the fiber amount, but also by the nature of the fine components in the cement mixture. Higher values for the adhesive strength of both substrates for brick (by 61%) and aerated concrete blocks (by 11%) were obtained for the plaster mortar with granulated slag (C9) compared with the fiber-cement mortar containing finely ground limestone (C8). It manifested in a greater depth of plaster layer failure, crack formation, and in greater cohesion damage between the substrate and mortar in the case of sample C9. The damage observed on both substrates was probably caused by the volume changes of the very finely ground granulated slag used in the proposed formulation (C9) because of its high tensile stress. The results of the comparative study of the physical and mechanical properties of the two cement plasters incorporated with recycled cellulosic fibers and a designed composition (a partial cement replacement by the ground materials—limestone and granulated blast furnace slag) provided complex information about their development. In addition, this study showed a future research direction towards optimizing the composition of the plaster mixture using waste material in order to better fix the plaster mortar to the substrate and to minimize its damage. A key aspect will also be to examine the long-term durability of the fiber-cement plaster mortar in terms of fiber degradation.

The results presented in this article demonstrate to researchers and designers the importance of fiber-cement plaster mortar composition and its influence on the properties of cement. It also highlights the benefits of these fiber-based plaster mortars for providing healthy living solutions, thanks to the fibers’ ability to regulate humidity inside buildings by absorbing and/or releasing water molecules, depending on the air conditions. 

## Figures and Tables

**Table 1:** Chemical composition of Portland cement CEM I 42.5 N.

Oxides/Element	Content (%)
MgO	0.81
Al_2_O_3_	2.13
SiO_2_	12.50
P_2_O_5_	0.35
SO_3_	2.86
Cl	0.09
K_2_O	0.80
CaO	49.79
TiO_2_	0.17
V_2_O_5_	0.02
MnO	0.03
Fe_2_O_3_	2.29
ZnO	0.02
SrO	0.04

**Table 2:** Chemical composition of waste paper cellulose fiber (WPF) samples.

Components of WPF (%)	Component Content (%) in WPF Samples		
	A	B	C
Holocellulose	71.03	71.98	81.30
Cellulose	47.40	46.95	56.97
Hemicellulose	23.63	25.03	24.33
Lignin	17.05	20.05	20.11
Ash	19.91	22.80	16.54
Extractives	1.39	1.69	1.72
Moisture	1.61	1.50	1.99

**Table 3:** Polymeric characteristics of cellulose in the WPF samples. DP—degree of cellulose polymerization; PDI—polydispersity index.

Cellulose Polymer Characteristic	WPF Samples		
	A	B	C
DP	1176	1190	1285
PDI	6.92	6.16	6.30

**Table 4:** Physical properties of WPF samples.

Characteristics of WPF Samples	WPF Sample		
	A	B	C
Density (kg/m^3^)	1843	1966	1943
Apparent density (kg/m^3^)	75	55	40
Max. fiber length (µm)	400	600	1200
Dry matter (%)	93	93	93
pH	7 ± 1	7 ± 1	7.5 ± 1
Average fiber width D (µm)	29.5	30.9	29.0
Average fiber length L (µm)	556	701	796
Aspect ratio L/D (-)	18.8	22.7	27.5
BET specific surface area (m^2^/g)	9.48	8.63	7.60
NLDFT cumulative pore volume (10^3^ cm^3^/g)	20.64	12.98	22.06
NLDFT pore radius (nm)	2.71	1.66	2.08
Thermal conductivity (W/m⋅K)	0.0634	0.0599	0.0595
Volume heat capacity ×10^−6^ (J/m^3^⋅K)	0.2097	0.1785	0.1709
Thermal diffusivity ×10^6^ (m^2^/s)	0.3024	0.3354	0.3478

**Table 5:** Chemical composition of the ground limestone and granulated slag.

Oxides/Elements	Limestone	Slag
	Content (%)	
MgO	0.59	0.29
Al_2_O_3_	0.15	4.17
SiO_2_	0.91	25.00
P_2_O_5_	0.18	0.52
SO_3_	0.07	1.04
Cl	0.01	0.02
CaO	61.26	35.70
K_2_O_3_	-	0.22
TiO_2_	0.01	0.22
V_2_O_5_	0.01	0.02
MnO	-	0.62
Cr_2_O_3_	0.10	-
Fe_2_O_3_	0.43	0.33
SrO	0.02	0.02

**Table 6:** Formulations for fiber-cement mortars.

Mortar Sample	C/WPF	W/C
Set I		
R1	-	0.50
R2	-	0.55
A1; B1; C1	500:1	0.55
A2; B2; C2	330:1	0.55
A3; B3; C3	200:1	0.55
Set II		
R3	-	0.75
A4; B4; C4	33:1	1.55
A5; B5; C5	17:1	1.55
A6; B6; C6	7:1	1.55
C7	3:1	1.55
Set III		
C8	6:1	2.08
	6:1	2.17

**Table 7:** Values of absorbency and capillary water absorption for 28-day hardened fiber-cement mortars.

C/WPFs	Mortar Sample	Absorbency (%)	Capillary Water Absorption (kg/m^2^·min ^0.5^)
	R2	8.66	0.23
500:1	A1	9.21	0.21
	B1	9.30	0.24
	C1	9.35	0.22
330:1	A2	9.61	0.25
	B2	10.25	0.23
	C2	10.35	0.24
200:1	A3	10.38	0.25
	B3	10.29	0.24
	C3	10.49	0.26
	R3	9.75	0.23
33:1	A4	11.41	0.26
	B4	12.24	0.36
	C4	12.62	0.38
17:1	A5	12.20	0.34
	B5	12.65	0.37
	C5	12.68	0.36
7:1	A6	12.82	0.38
	B6	12.91	0.39
	C6	12.75	0.37

**Table 8:** Properties of fresh fiber-cement mixtures, hardened mortars, and adhesive strength on two substrates of brick (B) and aerated concrete block (ACB).

Set III		
Property	C8	C9
Mean spill diameter (mm)	157	150
Bulk density (kg/m^3^)	1083	1112
Thermal conductivity coefficient (W/kg·m)	0.224	0.222
Absorbency (wt.%)	50.7	47.8
Capillary water absorption coefficient (kg/m^2^·min^0.5^)	2.71	1.59
Compressive strength (MPa)	4.86	10.07
Flexural strength (MPa)	1.70	3.32
Adhesive strength on brick (MPa)	0.215	0.347
Adhesive strength on aerated concrete block (MPa)	0.205	0.228

## Data Availability

The data presented in this study are available upon request from the corresponding author.
